# *In vitro* Dynamic Pharmacokinetic/Pharamcodynamic (PK/PD) study and CO_PD_ of Marbofloxacin against *Haemophilus parasuis*

**DOI:** 10.1186/s12917-015-0604-5

**Published:** 2015-12-01

**Authors:** Jian Sun, Xia Xiao, Rui-Juan Huang, Tao Yang, Yi Chen, Xi Fang, Ting Huang, Yu-Feng Zhou, Ya-Hong Liu

**Affiliations:** National Risk Assessment Laboratory for Antimicrobial Resistance of Animal Original Bacteria, South China Agricultural University, Guangzhou, 510642 China; Jiangsu Co-Innovation Centre for Prevention and Control of Important Animal Infectious Diseases and Zoonoses, Yangzhou, Jiangsu People’s Republic of China

**Keywords:** Marbofloxacin, PK/PD, *H. parasuis*, CO_PD_, Monte Carlo simulation

## Abstract

**Background:**

*Haemophilus parasuis* (*H. parasuis*) can invade the body and cause systemic infection under stress conditions. Marbofloxacin has been recommended for the treatment of swine infections. However, few studies have investigated the PK/PD characteristics and PK/PD cutoff (CO_PD_) of this drug against *H. parasuis*.

**Results:**

MICs of marbofloxacin against 198 *H. parasuis* isolates were determined. The MIC_50_ and MIC_90_ were 2 and 8 mg/L, respectively. An *in vitro* dynamic PK/PD model was established to study the PK/PD relationship of marbofloxacin against *H. parasuis*. The PK/PD surrogate markers C_max_/MIC, C_max_/MPC (the maximum concentration divided by MIC or mutant prevention concentration (MPC)) and AUC_24h_/MIC, AUC_24h_/MPC (the area under the curve during the first 24 h divided by MIC or MPC) simulated the antimicrobial effect of marbofloxacin successfully with the R^2^ of 0.9928 and 0.9911, respectively. The target values of 3-log_10_-unit and 4-log_10_-unit reduction for AUC_24h_/MPC were 33 and 42, while the same efficacy for AUC_24h_/MIC were 88 and 110. The CO_PD_ deduced from Monte Carlo simulation (MCS) for marbofloxacin against *H. parasuis* was 0.5 mg/L. The recommended dose of marbofloxacin against *H. parasuis* with MIC ≤ 2 mg/L was 16 mg/kg body weight (BW).

**Conclusions:**

The PK/PD surrogate markers AUC_24h_/MIC, C_max_/MIC and AUC_24h_/MPC, C_max_/MPC properly described the effects of marbofloxacin. Marbofloxacin can achieve the best efficacy at dosage of 16 mg/kg BW for strains with MIC values ≤ 2 mg/L, therefore, it is obligatory to know the sensitivity of the pathogen and to treat animals as early as possible. The very first CO_PD_ provide fundamental data for marbofloxacin breakpoint determination.

## Background

*Haemophilus parasuis* (*H. parasuis*) is not only a common inhabitant bacterium of the upper respiratory tract in swine but also an etiological agent of Glässer’s disease characterized by arthritis, fibrinous polyserositis, and meningitis [1]. *H. parasuis* can invade the body and cause systemic infection under stress conditions, for example, weaning, transporting, and decaying of maternal immunity [2]. It can also co-infect with immunosuppressive agents, *i.e.*, porcine reproductive and respiratory syndrome (PRRS) virus [2]. Strains of serovars 1, 5, 10, 12, 13, and 14 were highly virulent and caused death or morbidity [3]. Among all of the serovars, serovars 5 and 4 are the most prevalent among isolates reported in China [4], Denmark [5], Germany [3], and the United States [6, 7].

*H. parasuis* infection is often treated with sulfanilamide, quinolones, or cephalosporins. However, some isolates have developed resistance to these drugs [8]. The most important factor for the emergence and dissemination of resistance is the exposure, especially exposure to sub-optimal drug concentrations [9]. The PK/PD modelling which helps determine exposure-response relationships is of great importance in determining antimicrobial regimens administered to animals to attain appropriate effects [10]. The Fluoroquinolones and Cephalosporin of 3th and 4th generations that have been re-licensed in Europe took into account not only the classical paradigm of concentration-dependent dosage but also the PK/PD indices best descried the effects and minimized the emergence of resistances, such as AUC_24h_/MIC, C_max_/MIC, the percent time that drug concentrations were above the minimum inhibitory concentration (%T > MIC), AUC_24h_/MPC, the percent time that marbofloxacin concentrations were above the mutant prevention concentration (%T > MPC) or the mutant selection window (TMSW). Marbofloxacin, a third generation fluoroquinolone, has been developed solely for veterinary treatment. It acts as a concentration-dependent bactericidal agent against Gram-negative and gram-positive bacteria [11]. Marbofloxacin has been recommended by the European Committee and China for the treatment of swine infections with a dosage regimen of 2 mg/kg/24 h BW for three to five days. However, the PK/PD relationship of it against *H. parasuis* is sparse. Susceptibility breakpoint for an antimicrobial may assist in determining whether an antibacterial is potentially useful in the treatment of a bacterial infection. Knowing whether an antimicrobial is useful will promote prudent use of antimicrobial drugs. Breakpoints should be set prior to an antibacterial being used clinically or at the time of an approved use. Its setting requires integration of knowledge of the wild-type distribution of MICs, the PK/PD relationship of an antibacterial, and clinical outcomes of infections when the antibacterial is used [12]. Veterinary susceptibility breakpoints are developed by the Clinical and Laboratory Standards Institute (CLSI) subcommittee on Veterinary Antimicrobial Susceptibility Testing (VAST) [13]. At this time, however, no veterinary specific clinical breakpoints of marbofloxacin have been established for swine disease caused by *H. parasuis*.

CO_PD_ determined by MCS that considers pharmacokinetic variation in target animals and PK/PD indices assisted in the defining of susceptibility breakpoints from the perspective of exposure–response relationship [14]. This method has also been used by regulatory agencies such as the U.S. FDA and the European Medicines Agency (EMA), or relevant specialized groups such as CLSI-AST and the European Committee on Antimicrobial Susceptibility Testing (EUCAST), in defining the susceptibility breakpoints [15].

The purpose of this investigation was to study the PK/PD relationship of marbofloxacin against *H. parasuis*, derive a CO_PD_ of marbofloxacin and recommend a reasonable dosage regimen. MICs of marbofloxacin against those isolates were determined. An *in vitro* PK/PD infection model was used to investigate marbofloxacin effects against *H. parasuis* strain of serotype 5, which is a highly virulent serotype and is one of the most prevalent serotypes in China. Pharmacokinetics of marbofloxacin in swine obtained from a previous study and PK/PD indices were integrated into a Monte Carlo simulation to derive a CO_PD_. A rational regimen of marbofloxacin against *H. parasuis* was determined.

## Methods

### Animal ethics

All husbandry practices and experimental operations were performed with full consideration of animal welfare. Research ethical approval was granted by the South China Agriculture University Animal ethics committee (2014–03).

### Strains and antibiotic

A strain of *H. parasuis*, serovar 5 (V5), kindly provided by Professor Ming Liao, College of Veterinary Medicine, South China Agricultural University, Guangzhou, Guangdong Province, China was used in the present study. V5 was marbofloxacin susceptible with a MIC of 0.015 mg/L. The other 189 isolates of *H. parasuis* were collected from swine in south China regions between August 2010 and July 2011. Serotypes of these isolates are not known. Tryptone soya agar (TSA) and Tryptone soya broth (Oxoid Ltd., Basingstoke, Hampshire, UK), supplemented with 2 % beta-Nicotinamide adenine dinucleotide trihydrate (NAD) (Qingdao Hope Bio-Technology Co., Ltd., Shandong, China) and 5 % new-born calf serum (Guangzhou Ruite Bio-tec Co., Ltd., Guangdong, China), were used to culture *H. parasuis*. Marbofloxacin was purchased from Hebei Yuanzheng Pharmaceutical Company (Hubei, China).

### Susceptibility testing

Considering that there is no recommended testing method by CLSI’s VAST Subcommittee for *H. parasuis*, MICs were conducted in accordance with the CLSI recommendations for *Actinobacillus pleuropneumoniae*. [16] (Wakiec et al., 2008) The *Actinobacillus pleuropneumoniae* ATCC 27090 strain was used for quality control purpose. MIC_50_ and MIC_90_ were defined in the present study as the lowest concentration that inhibited the growth of 50 % and 90 % of isolates tested, respectively. The mutant prevention concentration (MPC) value was determined according to previous reports [17, 18]. Single bacteria colony from 24 h growth on TSA was grown for 12 h in TSB broth, then concentrated through centrifugation and re-suspended it in TSB to a final concentration of ~ 3 × 10^10^ CFU/mL. An aliquot of 500 μL samples was plated onto TSA plates containing various concentrations of marbofloxacin, and then incubated for 24 h, 36 h and 48 h for re-growth. MPC_pr_ was defined as the lowest drug concentration that inhibits growth. A second measurement was performed using linear drug concentration increment within 20 % per sequential increase. All the determinations were carried out in triplicates.

### In vitro PK/PD model

The *in vitro* one-compartment PK/PD infection model equipment was constructed according to previously described method with some improvements [19]. An inverted 50 mL centrifuge tube with a cellulose ester membrane (0.2-μm pore size) covering the top was placed in the central compartment to prevent bacteria from flowing out to the medium. A magnetic stir bar was placed on the bottom of the central compartment which mixed the broth and enabled the drug to fully contact the bacteria. The flow rate was 0.171 mL/min to simulate the half-life of marbofloxacin in swine as described previously [20].

### In vitro time kill curves of marbofloxacin

A 12 h culture of V5 at logarithmic phase was added to the central compartment to reach a final concentration of 10^7^ colony forming unit (cfu)/mL. An incubation period of 30 min was applied to adapt the bacteria to the new environment. Different doses of marbofloxacin or control (sterile normal saline) were administered into the central compartment, and at the same time, the peristaltic pump was turned on. Samples were obtained at time points of 0, 3, 6, 9, 12, and 24 h. 100 μL of the samples were diluted properly with sterile normal saline, aliquots of the last four diluted samples were dropped onto the TSA plates and incubated at 37 °C for 24 h. The limit of determination was 400 cfu/mL.

### Pharmacokinetics and PK/PD analysis

The samples for marbofloxacin concentration determination were centrifuged at 8,000 rpm at 4°C for 10 min. The supernatant was stored at −80 °C and analyzed using High Performance Liquid Chromatography (HPLC), which had been optimized by our laboratory [21] within 1 month. All experiments were performed in duplicate on different days. The PK data were analyzed using Phoenix WinNonlin 6.0 software (Pharsight Co. Ltd.).

As marbofloxacin is concentration dependent, the PK/PD index of marbofloxacin was AUC_24h_/MIC and C_max_/MIC [22]. The PK/PD indexes were calculated using the pharmacokinetic data and MIC value in each dose of the time–kill curve. The *in vitro* drug effect was quantified by changes in log_10_ cfu counts between 24 h and 0 h. Data were analysed using sigmoid *E*_max_ model WINNONLIN software (version 6.1; Pharsight, CA, USA) with the following equation:

$$ E={E}_0+\frac{E_{\max}\times {C}_e^N}{E{C}_{50}^N+{C}_e^N} $$

Where *E*_0_ is the change in log_10_ cfu/mL after 24 h incubation in the control sample, compared with the initial inoculum. E_max_ is the difference in effect between the greatest amount of growth (as seen for the growth control, E_0_) and the greatest amount of kill. *C*_e_ is the AUC_24h_/MIC, C_max_/MIC or C_max_/MPC in the effect compartment. EC_50_ is the AUC_24h_/MIC, C_max_/MIC or C_max_/MPC value producing a 50 % reduction in bacterial counts from the initial inoculum, and *N* is the Hill coefficient that describes the steepness of the curve.

### Monte Carlo analysis (MCS)

A 10,000-subject Monte Carlo simulation was conducted using Crystal Ball Professional V7.2.2 software based on a previous pharmacokinetic study of marbofloxacin in pigs and PK/PD target indices obtained in this study. AUC_24h_ and C_max_ were assumed to be log-normally distributed in the form of mean values and confidence intervals. The CO_PD_ is the MIC at which the probability of target attainment (PTA) equals to 90 %, which is the most commonly used standard for susceptibility breakpoints in other bacteria [12].

### Dosage calculation

In order to deduce a more rational regimen, the general formula was employed to estimate dosages for different magnitudes of efficiency [23].

$$ \mathrm{Dose}=\frac{a\times \frac{{\mathrm{AUC}}_{24h}}{\mathrm{MC}}\times {\mathrm{MC}}_{90}}{F\times \mathrm{f}\mathrm{u}\times 24\mathrm{h}} $$

Where Dose is the optimal dose (mg/kg day), CL is the body clearance (L/kg day), AUC/MIC is the breakpoint marker for the desired effect, MIC_90_ is the MIC inhibiting 90 % of strains (mg/L), F is the bioavailability, and fu is the free drug fraction.

## Results

**Fig. 1:**
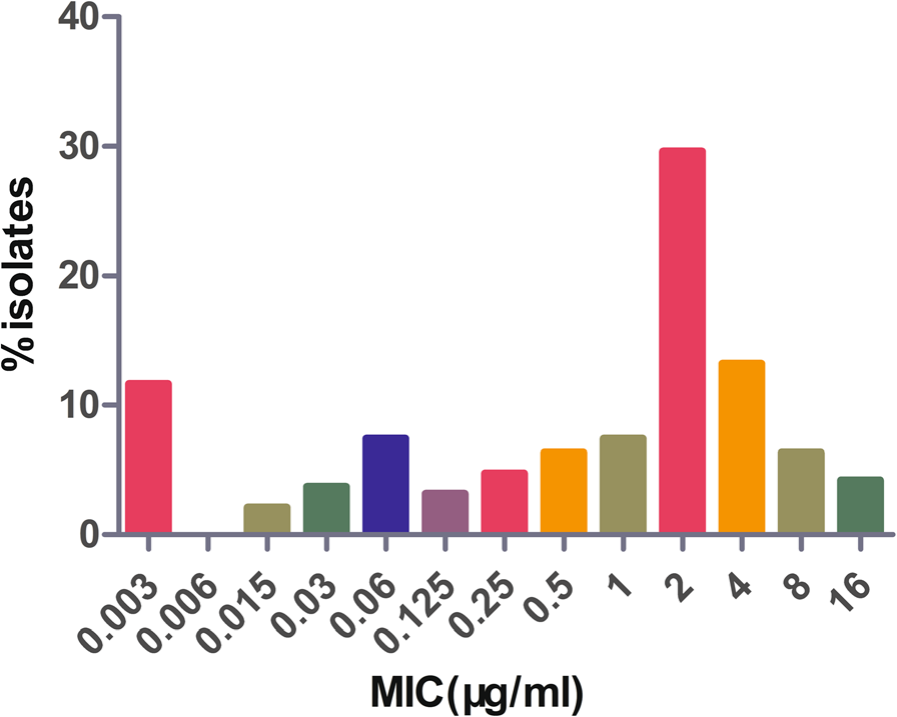
Minimum inhibitory concentrations of marbofloxacin against 199 isolates of *H. parasuis*

MICs of marbofloxacin against *H. parasuis* were widely distributed, ranging from 0.003 mg/L to 16 mg/L (Fig. 1). A trimodal distribution was observed with peak value observed at 0.003, 0.06, and 2 mg/L, respectively. The MIC_50_ and MIC_90_ were 2 and 8 mg/L, respectively. The MPC of strain V5 was 0.04 mg/L at different time endpoints (24, 36 and 48 h), which was about 2 ~ 3 times of the MIC value (0.015 mg/L).

**Fig. 2:**
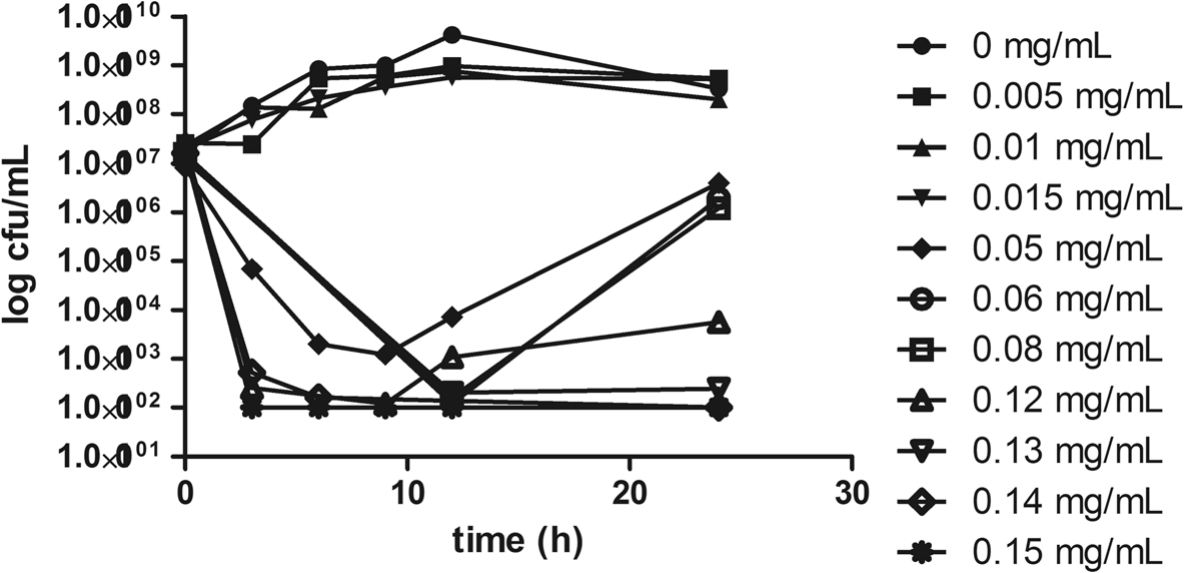
Time–kill curve of marbofloxacin against *H. parasuis* in the *in vitro* PK/PD model

**Fig. 3:**
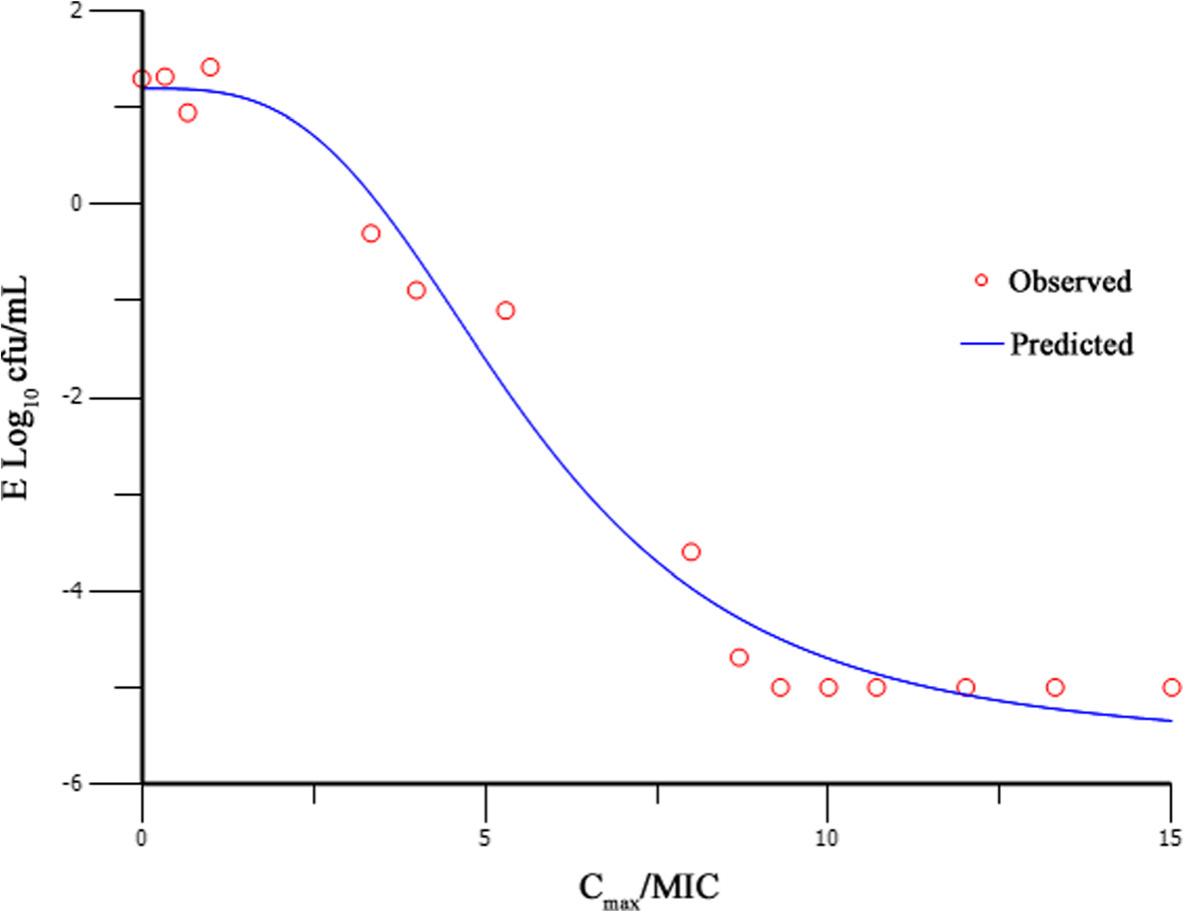
Sigmoid *E*_max_ model relationships between antibacterial effect [E, log_10_ (cfu/mL)] and C_max_/MIC of marbofloxacin in the *in vitro* PK/PD model against *H. parasuis* with an inoculum size of 1 × 10^7^ cfu/mL

**Fig. 4:**
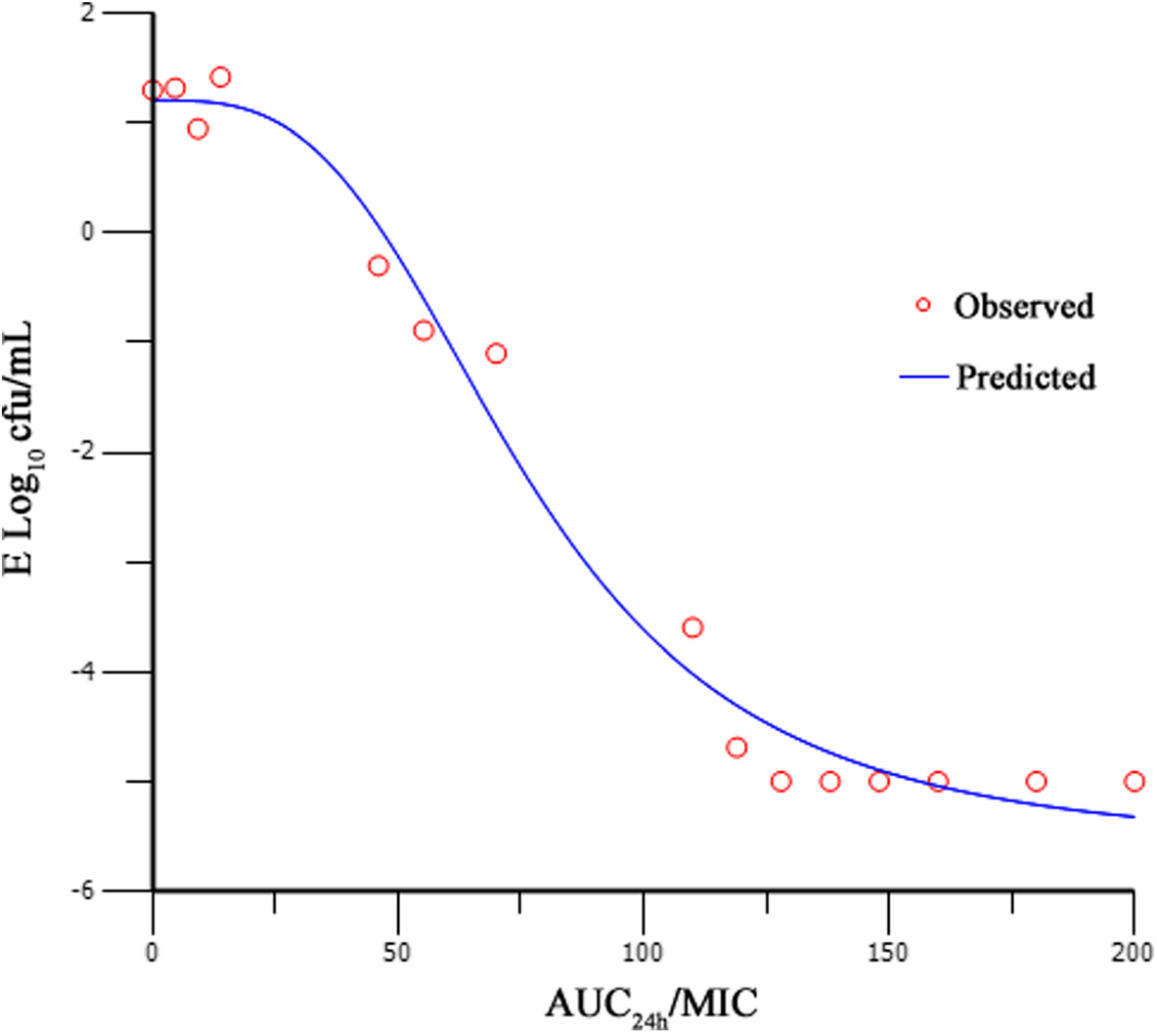
Sigmoid *E*_max_ model relationships between antibacterial effect [E, log_10_ (cfu/mL)] and AUC_24h_/MIC of marbofloxacin in the *in vitro* PK/PD model against *H. parasuis* with an inoculum size of 1 × 10^7^ cfu/mL

The pharmacokinetics of marbofloxacin in pigs was well simulated by this model with the relative deviation below 7 %. Time killing curves were shown in Fig. 2. The marbofloxacin inhibited *H. parasuis* moderately when the AUC_24h_/MIC was less than 13.8. The bacteria decreased rapidly within 12 h, but re-grew to 10^6^ cfu/mL at 24 h with the AUC_24h_/MIC of 46. When the AUC_24h_/MIC was 55 and 73, *H. parasuis* could not be detected at 12 h, however, re-growth was observed at 24 h. When AUC_24h_/MIC was 92 and 110, marbofloxacin killed *H. parasuis* without regrowth in 24 h (4-log-unit and 5-log-unit decrease, respectively). The relationship between *in vitro* antimicrobial efficacy and PK/PD surrogate markers (AUC_24h_/MIC or C_max_/MIC) was described using the sigmoid *E*_*max*_ model. Both of the PK/PD surrogate markers simulated the *in vitro* antimicrobial effect of marbofloxacin successfully with the R^2^ of 0.9928 and 0.9911 respectively (Figs 3, 4). The estimated Log *E*_*max*_, Log *E*_*0*_, EC_50,_ and slope were shown in Tables 1 and 2 respectively. The target values of 3-log_10_-unit and 4-log_10_-unit decreases for C_max_/MIC were 6.5 and 8 while for AUC_24h_/MIC were 88 and 110, respectively. The same effects for surrogates C_max_/MPC and AUC_24h_/MPC were 2.5, 3 and 33, 42 respectively. They also simulated the *in vitro* antimicrobial effect of marbofloxacin successfully with the R^2^ of 0.9928 and 0.9911 respectively.

**Table 1:** PK/PD analysis of marbofloxacin with the parameter of C_max_/MIC and C_max_/MPC against *H. parasuis*

Parameter (units)	Value
Log E_max_ (cfu/mL)	6.8
Log E_0_ (cfu/mL)	1.2
C_max_/MIC EC_50_	5.6
C_max_/MIC (bacteristasis)	3
C_max_/MIC (bactericidal)	6.5
C_max_/MIC (bacteria elimination)	8
C_max_/MPC (bactericidal)	2.5
C_max_/MPC (bacteria elimination)	3
Slope (N)	3.2

Note: *E*_0_ is the change in log_10_ cfu/mL after 24 h incubation in the control sample compared with the initial inoculum. E_max_ is the difference in effect between the greatest amount of growth (as seen for the growth control, E_0_) and the greatest amount of kill. EC_50_ is the C_max_/MIC value producing a 50 % reduction in bacterial counts from the initial inoculum, and *N* is the Hill coefficient that describes the steepness of the dose–response curve

**Table 2:** PK/PD analysis of marbofloxacin with the parameter of AUC_24h_/MIC and AUC_24h_/MPC against *H. parasuis*

Parameter (units)	Value
Log E_max_ (cfu/mL)	6.8
Log E_0_ (cfu/mL)	1.2
AUC_24h_/MIC EC_50_	76
AUC_24h_/MIC (bacteristasis)	47
AUC_24h_/MIC (bactericidal)	88
AUC_24h_/MIC (bacteria elimination)	110
AUC_24h_/MPC (bactericidal)	33
AUC_24h_/MPC (bacteria elimination)	42
Slope (N)	3.2

Note: *E*_0_ is the change in log_10_ cfu/mL after 24 h incubation in the control sample compared with the initial inoculum. E_max_ is the difference in effect between the greatest amount of growth (as seen for the growth control, E_0_) and the greatest amount of kill. EC_50_ is the AUC_24h_/MIC value producing a 50 % reduction in bacterial counts from the initial inoculum, and *N* is the Hill coefficient that describes the steepness of the dose–response curve

**Fig. 5:**
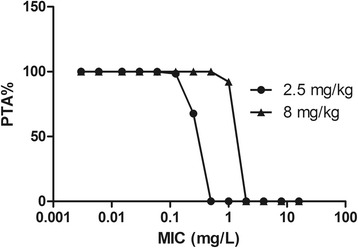
The Probability of target attainment (PTA) for different marbofloxacin doses against isolates of *H. parasuis* with different MICs

As there are no PK data of marbofloxacin with a dose of 2 mg/kg BW, data of 2.5 mg/kg BW were used for MCS. In the simulation, with the PK/PD target AUC_24h_/MIC of 88, PTA > 90 % could only be achieved for isolates with MIC ≤ 0.125 mg/L (Fig. 5). For dosage regimen of 8 mg/kg BW with a single dose administered by IM, PTA > 90 % could be achieved for isolates with MIC ≤ 0.5 mg/L (Fig. 5). The CO_PD_ for marbofloxacin against *H. parasuis* was 0.5 mg/L. The recommended dose of marbofloxacin for bactericidal effect against *H. parasuis* was 16 mg/kg BW.

## Discussion

Currently, a great deal of information is available on the PK/PD relationship of fluoroquinolones. The parameters C_max_/MIC and AUC_24h_/MIC correlate well with therapeutic outcome. According to literature, AUC_24h_/MIC of >125 h and C_max_/MIC of >10 was usually used as a threshold for successful therapeutic outcome of fluoroquinolones against gram negative bacteria [23]. Nevertheless, these thresholds may be different for some fluoroquinolones. The greatest influence for the differences was the immune status of the animal. Furthermore, the PK/PD indices of the same drug against different pathogens also vary. For example, the threshold of AUC_24h_/MIC is 46 h for bactericidal action in an *ex vivo* PK/PD study of marbofloxacin against *Mannheimia haemolytica* [24]; AUC_24h_/MIC ratios for no reduction, 3 log10 and 4 log10 reductions in bacterial count from the initial inoculum count were 41.9, 59.5, and 68.0 h for *M. haemolytica* and 48.6, 64.9, and 74.8 h for *P. multocida* in an *ex vivo* PK/PD study of marbofloxacin [25]. So it is of great importance to study the PK/PD indices of fluoroquinolones individually. Data on the PK/PD indices of marbofloxacin against *H. parasuis* are limited. In this study, PK/PD surrogates (C_max_/MIC, AUC_24h_/MIC and C_max_/MPC, AUC_24h_/MPC) simulated the bacterial reduction effects very well. The AUC_24h_/MIC ratios for no reduction, 3 log10, and 4 log10 reductions in bacterial count were 50, 88, and 110 h, while the C_max_/MIC ratios for those effects were 3.5, 6.5, and 8. The threshold value is higher than those derived from *ex vivo* PK/PD model. For these comparisons, it should be noted that there are significant differences between dynamic *in vitro* and *ex vivo* conditions. There is a continuous exposure to a fixed concentration of the agent for a defined duration (e.g., 24 h) in an *ex vivo* model, whereas a gradient of concentration in the dynamic *in vitro* model. The level of AUC_24h_/MPC (33 for 3 log_10_ reductions) was higher than that (12.89 for bactericidal effect) resulted from a tissue cage model of marbofloxacin against *Pasteurella multocida* [26]. The greatest influence for the differences might be the immune status of the animal. The value of C_max_/MPC (2.5 for bactericidal effect) was almost the same with that (C_max_/MPC > 2.2) of levofloxacin against *staphylococcus aureus* in a hollow fiber PK/PD model [27]. Though, both the PK/PD surrogates derived from MIC and MPC described the effect properly, PK/PD surrogates derived from MPC has been proven to be superior for fluoroquinolones over the classic PK/PD indices based on MIC for minimizing the emergence of resistances and preventing therapeutic failure [27].

Susceptibility breakpoint setting requires knowledge of the wild-type distribution of MICs, assessment of the PK/PD indices, and study of the clinical outcome of infections treated with the antibacterial. Monte Carlo simulation shows great advantage in supporting determination of the susceptibility breakpoint using drug exposure-effect relationship [14], which takes pharmacokinetic variation and PK/PD indices into consideration. For the simulation, as it had been proved that the efficacy of a single dosing regimen was better than doses administered every 24 h or 48 h of the same total amount of marbofloxacin [26], the EMA recommended dose regimen can be converted to 8 mg/kg BW with a single dose. Though both AUC_24h_/MIC and C_max_/MIC were PK/PD indices of fluoroquinolones, AUC_24h_ is a much more robust estimation than the one of a single snapshot C_max_ which depends on many factors such as sampling schedule. Therefore the PK/PD target was defined to be AUC_24h_/MIC with value of 88. The CO_PD_ of marbofloxacin against *H. parasuis* was 0.5 mg/L under one short dose of 8 mg/kg. This CO_PD_ value was equal to the clinical breakpoints values and the PK/PD breakpoints values of ciprofloxacin, moxifloxacin (0.5 mg/L), ofloxacin (0.5 mg/L), and levofloxacin(1 mg/L) against *Haemophilus influenzae,* another bacteria of *Haemophilus spp* in human clinic use [28]*.* Unfortunately, the proposed PK/PD cutoff would designate a large proportion of clinical *H. parasuis* isolates as resistant to the marbofloxacin. Swine infected by these isolates will probably have a low likelihood of responding to therapy.

Clinical dosage regimens of antimicrobial agents are traditionally determined by relating the PK of drugs in healthy animals and the *in vitro* antibacterial activity or the treatment outcome at given dosages in disease models or in clinical trials involving limited strains. Although these parameters can predict the potency of the drug against pathogens to a certain extent, they actually don’t provide information on the time course of antimicrobial activity. Fortunately, the relationship of PK/PD parameters and the clinical outcomes have been fully investigated and applied in optimizing drug regimens; the regimens based on PK/PD results were usually calculated by the general formula [23]. In the calculation, the PK/PD threshold, the PK parameters and MIC distribution were taken into accounted. As the *fu* of marbofloxacin in swine plasma was sparse, the value in dog plasma was used to substitute that. According to our best knowledge, the CL of marbofloxacin was 0.065 ± 0.011 L/kgh; the bioavailability was 90 % ± 28 %; the protein binding rate was 21.81 % ± 6.26 %; MIC_50_ is 2 mg/L [20, 29]. When the MIC_90_ was used to calculate the dose, we found the result (64 mg/kg BW) was too high to apply in clinical usage. Considering the MICs of some *H. parasuis* isolated in China are very high, they may exhibit resistance to marbofloxacin that cannot be treated by marbofloxacin. MIC_50_ was used in the calculation. the result showed that a dose of 16 mg/kg would be able to achieve bactericidal effect. This dose is much higher than the recommended dose (2 mg/kg for 3 to 5 days), and higher than that (10 mg/kg) [30] recommended for bovine respiratory disease based on the theoretical principle of single-injection, short-acting antibiotic (SISAAB), which has been primarily developed and applied to fluoroquinolones used in human medicine [31]. It seems that even a dose as high as 16 mg/kg BW cannot cure all the *H. parasuis* infection in China. It is well-known that fluoroquinolones can lead to cross-resistance among different members of the class [32], since they are widely used to treat respiratory diseases. It is better to check the susceptibility of pathogens before drug administration, as resistance determinants may transfer to human pathogenic bacteria, resulting in the failure of antibiotics in treatment of bacterial infection.

However, there are some limitations in our study. First, the PK/PD indices targets were based on the drug concentration at infection sites, whist drug concentration of plasma were used for MCS. Though, the penetration of marbofloxacin is good and the tissue concentration is similar to that in blood [26], concentration at infection sites should be simulated in future trials. A second limitation is that the recommended regimen is useful in just the regions of China as MIC probability distribution of a determined pathogen may vary between countries and regions and even time. Finally, our proposed CO_PD_ will need to be validated in the clinical outcome.

## Conclusions

In summary, this study established an *in vitro* dynamic PK/PD modelling of marbofloxacin against *H. parasuis*. The target PK/PD values of marbofloxacin for 3-log_10_-unit and 4-log_10_-unit decreases effects were C_max_/MIC of 6.5 and 8, C_max_/MPC of 2.5 and 3, AUC_24h_/MIC of 88 and 110 or AUC_24h_/MPC of 33 and 42 respectively. The very first marbofloxacin CO_PD_ (0.5 mg/L) derived based on MCS was of great utility in marbofloxacin susceptibility test and dosing design. Marbofloxacin can have the best efficacy at dosage of 16 mg/kg BW for strains with MIC values ≤ 2 mg/L, therefore, it is obligatory to know the sensitivity of the pathogen and to treat animals as early as possible.

## Abbreviations

H. parasuis: Haemophilus parasuis
PK/PD: Pharmacokinetic/pharamcodynamic
CO_PD_: Pharmacokinetic/pharamcodynamic cutoff
MICs: Minimal inhibitory concentrations
C_max_/MIC: The maximum concentration divided by MIC
UC_24h_/MIC: The area under the curve during the first 24 h divided by the minimum inhibitory concentration
MPC: Mutant prevention concentration
C_max_/MPC: The maximum concentration divided by mutant prevention concentration
AUC_24h_/MPC: The area under the curve during the first 24 h divided by mutant prevention concentration
MCS: Monte Carlo simulation
BW: Body weight
PRRS: Porcine reproductive and respiratory syndrome
%T > MIC: The percent time that marbofloxacin concentrations were above the minimum inhibitory concentration
%T > MPC: The percent time that marbofloxacin concentrations were above the mutant prevention concentration
TMSW: The mutant selection window
VAST: Veterinary Antimicrobial Susceptibility Testing
EMA: European Medicines Agency
CLSI: The Clinical and Laboratory Standards Institute
EUCAST: The European Committee on Antimicrobial Susceptibility Testing
V5: Serovar 5
TSA: Tryptone soya agar
NAD: Beta-Nicotinamide adenine dinucleotide trihydrate
MIC_50_ and MIC_90_: MIC, inhibiting the growth of at least 50 % and 90 % of isolates in a test population
OD: Optical density
Cfu: Colony forming unit
